# Effect of Mindfulness-Based Stress Reduction on the Well-Being, Burnout and Stress of Italian Healthcare Professionals during the COVID-19 Pandemic

**DOI:** 10.3390/jcm11113136

**Published:** 2022-05-31

**Authors:** Marco Marotta, Francesca Gorini, Alessandra Parlanti, Sergio Berti, Cristina Vassalle

**Affiliations:** 1 Fondazione CNR-Regione Toscana G. Monasterio, 54100 Massa, Italy; marco.marotta@ftgm.it (M.M.); alessandra.parlanti@ftgm.it (A.P.); berti@ftgm.it (S.B.); 2 Institute of Clinical Physiology, National Research Council, 56124 Pisa, Italy; fgorini@ifc.cnr.it; 3 Fondazione CNR-Regione Toscana G. Monasterio, Via Moruzzi 1, 56124 Pisa, Italy

**Keywords:** COVID-19, mindfulness, healthcare professionals, psycho-emotional status, distress, PGWBI, PSS, MBI, FCV-19S

## Abstract

COVID-19 has overwhelmed healthcare systems and increased workload and distress in healthcare professionals (HCPs). The objective of this study was to evaluate baseline distress before and after the pandemic, and the effect of Mindfulness-Based Stress Reduction (MBSR) training on well-being (PGWBI), stress (PSS) and burnout (MBI) in Italian HCPs. Moreover, the “fear of COVID-19” (FCV-19S) questionnaire was administered to HCPs participating in the post-emergency MBSR program. Baseline distress results were moderate in all groups. No differences between baseline distress were observed between the groups of HCPs beginning the MBSR courses in the pre or post pandemic period. Total PGWBI lowered with aging. Additionally, FCV-19S positively correlated with age. MBSR was able to lower distress levels, except for depersonalization, which increased, while emotional exhaustion decreased in the group enrolled in the last post-pandemic MBSR course. Levels of fear of COVID-19 in HCPs significantly decreased after MBSR training. The lack of change in baseline distress over time indicates that it is more influenced by work-related distress than by the pandemic in our HCPs. In view of its beneficial effects on psycho-emotional status, MBSR training may represent an effective strategy to reduce distress in emergency periods as well as an essential part of HCPs’ general training.

## 1. Introduction

Health care professionals (HCPs) are typically under pressure of great stress given the nature of their work and environment. Although their contribution was critical in facing the global challenge related to COVID-19, HCPs are at a higher risk of stress-related problems/disorders, such as anxiety, depression, and insomnia, during the pandemic (estimated in a recent meta-analysis with a prevalence rate of 23.2, 22.8 and 38.9%, respectively [1]), for many reasons, including a high fear of infecting themselves or relatives, growing patient load, lack of adequate equipment, prolonged shift work, etc. Accordingly, a recent review evidenced the increase in depression/depressive symptoms (six studies) and anxiety (eight studies) among HCPs [2]. Results from another systematic review and meta-analysis on the psychological and mental burden of COVID-19 among HCPs, the general population, and patients with higher COVID-19 risk (including 62 studies from 17 countries published between 1 November 2019 to 25 May 2020), suggested that the prevalence of anxiety and depression appeared to be similar between HCPs and the general population, although some countries, including Italy, reported a higher-than-pooled prevalence among HCPs [3]. In particular, the highest prevalence of anxiety was observed in Italy both in the general population (81% (95% confidence interval: 80–83%)) and among HCPs (57% (52–63%)) [3]. Additionally, in the general population, the highest prevalence of depression was observed in Italy (67% (65–69%)), although unfortunately no study evaluating the prevalence of depression in Italian HCPs was included in the analysis [3]. Another survey conducted on 145 Italian HCPs during March-April 2020 revealed that those who work in COVID-19 wards exhibited higher levels of both depressive and post-traumatic stress symptoms than those working in other healthcare units [4]. Approximately 50% of 2195 HCPs working in intensive care units, in sub-intensive COVID-19 units and in other front-line services, who participated in a web-based survey from April to May 2020, showed symptoms of post-traumatic distress and of clinically relevant anxiety [5]. On the other hand, a cross-sectional, multicentre study carried out in 77 hospitals in France in 2021 found that about 57% of HCPs suffered from psychological distress, but only 21% showed symptoms of potential post-traumatic stress [6]. Indeed, variations across countries in the scores for depression, anxiety, and stress are likely the consequence of various factors, such as the different number of COVID-19 cases and fatality rates, different pressures on healthcare systems, or the pandemic peaking at different times [7]. To note, Italy was the first European country to undergo the pandemic, and the healthcare system had little time to take preventive action and organize support interventions. Thus, it is not surprising that Italian HCPs in particular experienced elevated distress levels.

Mindfulness-based stress reduction (MBSR), originally proposed to face chronic diseases by reducing distress and improving quality of life in such patients, has become an increasingly prevalent psychological strategy worldwide. Also applied in HCPs, a series of systematic reviews and meta-analyses has documented the effectiveness of MBSR in reducing HCP anxiety, depression and stress, and increasing mindfulness, mood, resilience, self-compassion, self-efficacy, and empathy [8,9,10,11,12]. Therefore, mindfulness-based interventions are strongly recommended by health and educational organizations to implement stress management and enhance the psychosocial well-being of HCPs [12].

Recently, after the onset of COVID-19, mind–body practices have been proposed and evaluated to improve the psycho-emotional status of HCPs, including perceived stress, demonstrating their acceptance by HCPs, feasibility and effectiveness [13,14]. Since professionals with low levels of job satisfaction are more likely to experience distress (e.g., anxiety and depression), a supportive work environment is crucial to promote resilience and job satisfaction, providing the highest standard of care in the safest possible environment, especially during an emergency such as COVID-19 [15,16]. Indeed, MBSR can help HCPs to improve interpersonal relationships with patients and colleagues, to take better care of themselves and others, to pay more attention to the present moment, developing concentration and consequently improving work performance, being more available to listen and more motivated. All of these advantages can largely offset the limited costs of setting up permanent periodic courses in the hospitals, where MBSR training may serve as one of the effective tools in times of emergency, such as a pandemic, as well as an essential part of HCPs’ general training.

To the best of our knowledge there are no published studies evaluating the effect of MBSR on levels of distress by using a battery of questionnaires to assess psycho-emotional status in HCPs belonging to the same workplace before and after the pandemic onset. Hence, we aimed to evaluate distress levels of HCPs before and after the pandemic, evaluated by the Psychological General Well-Being Index (PGWBI), the Perceived Stress Scale (PSS), the Maslach Burnout Inventory (MBI), and the effect of a mindfulness intervention held before and in different periods during the pandemic. Moreover, the “fear of COVID-19” questionnaire was administered to HCPs enrolled after the spread of SARS-CoV-2 infection and participating in the last MBSR training course.

## 2. Materials and Methods

### 2.1. Study Sample and Procedures

This study is a cross-sectional survey. Data collection took place between 8 October 2019 and 22 April 2021. Fondazione Monasterio (FTGM) is a cardiological centre of excellence in Tuscany with two hospitals in Massa and Pisa, Italy. These hospitals were not COVID-19-dedicated hospitals at the time of observation, but two major cardiovascular centres serving a vast territory of northwestern Tuscany.

FTGM periodically organizes 8-week Mindfulness-Oriented Meditation Training courses as a part of a preventive medicine project to support HCPs’ mental health (e.g., to prevent professional burnout and improve well-being). All the courses were held by one of the authors (M.M.), a psychologist with more than five years of experience in practicing and teaching mindfulness meditation, in a quiet, heated classroom, face-to-face. Participation was voluntary, and anonymity was guaranteed. Data were also collected on the gender, age and occupational category of the participants. Written informed consent was obtained prior to participation.

**Figure 1:**
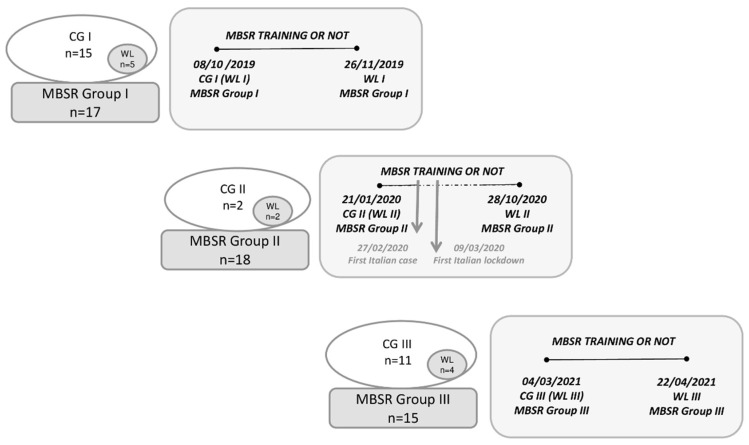
Timeline of the study, representing the different experimental and waiting list groups along with study measures. Abbreviations: CG: control group; MBSR: Mindfulness-Based Stress Reduction course; WL: waiting list.

A total of 50 health front-line professionals (mean age 44.4 ± 10.5, 42 women) including doctors, nurses and similar HCPs from the two FTGM hospitals in Pisa and Massa, took part in the MBSR courses (experimental group), 17 subjects in the pre-COVID period (Group I: 8 October 2019–26 November 2019), 18 started in the pre-COVID period and ended in the post-COVID period (Group II: on 21 January 2020 the course was interrupted due to the pandemic and resumed in September 2020, ended on 28 October 2020), and 15 took the course entirely in the post-COVID period (Group III: 4 March 2021–22 April 2021) (timeline reported in Figure 1). The questionnaires were administered before (T0) and after (T1) the training course. The control subjects were defined as subjects who filled out the questionnaires only at baseline (n = 28, mean age 42.0 ± 12.2, 23 women). For each session, a waiting list (WL) group was created among the controls, with participants in this group completing the questionnaires in parallel with those of the experimental group (Figure 1). The subjects of the WL group participated in the subsequent MBSR course. The study protocol was approved by the local Ethics Committee (number 19214, 11 February 2021).

### 2.2. Mindfulness-Based Stress Reduction Course

The MBSR technique was proposed by Kabat-Zinn in the 1970s, mixing mindfulness meditation, body awareness, yoga and deepening behavior, thinking, feeling and action. Dr Kabat-Zinn described mindfulness as “awareness derived by paying attention on purpose, in the present moment, non-judgmentally” [17]. The MBSR course is a common mindfulness meditation training program of 8 weeks, with a 2 h group meeting every week and recommended 30 min daily meditation practice at home. Its structure is divided into three activities: teaching on topics related to meditative practice, guided practice and a final stage of sharing experiences and asking the instructor questions. Topics covered in the teaching embrace a historical introduction to mindfulness meditation, what mindfulness meditation is and how to practice this discipline, coping with discomfort with a mindful attitude, attention and awareness, being in the here and now, disidentification, deautomatization, and letting go. Each weekly session began with a brief presentation of the session theme, followed by related guided experiential exercises. An MBSR lesson includes exercises as follows:

- focus on the breath;
- meditation on an object of interest for the person, focusing on its shape, size, color and smell;
- mindful eating, focusing on a food, involving all the senses: smell, taste, touch, sight, and even sound;
- attention paid to the sensations while walking on and when the feet touch the floor;
- training in formal mindfulness meditation techniques involving simple stretches and postures (Mindfulness Yoga) and “Bodyscan” (a meditative practice increasing body awareness and sensations in a gradual mental scan from the feet to the head).

Participants were given opportunities to discuss the material and ask questions, and experiential homework was advised. The instructor (M.M.) was available for any problems that emerged from the participants before the start of the lessons.

### 2.3. Assessments/Questionnaires

HCPs were given the Italian version of validated questionnaires described thereinafter, which are also commonly used in HCP cohorts [18,19,20,21].

The Psychological General Well-Being Index (PGWBI) is a 22-item self-reported questionnaire to measure the level of subjective psychological well-being or discomfort in the previous 4 weeks [22]. The items are rated on a 6-point scale (Likert scale from 0 to 5), and explore six different dimensions: anxiety (5, 8, 17, 19, 22 items), depressed mood (3, 7, 11), positive well-being (1, 9, 15, 20), self-control (4, 14, 18), general health (2, 10, 13) and vitality (6, 12, 16, 21), where a high score is indicative of elevated levels of psychological well-being. The total scores can reach a maximum value of 110 points. Global scores below 54 points reflect severe distress, between 55 and 65 moderate distress, and between 66 and 100 indicate a positive PGWBI or “no distress” status [22].

The Perceived Stress Scale (PSS) is a self-reported 10-item scale that evaluates the perceived intensity of stress over the previous 4 weeks (global perceived stress–PS) [23]. Each item is rated on a 5-point Likert scale (from 0-never to 4-very often). The scale values range from 0 to 40, with a higher score indicating a higher degree of subjective stress. Total scores 0–13 would be considered low PS, 14–26 as moderate PS, and 27–40 as high PS [24].

The Maslach Burnout Inventory (MBI) measures burnout by using a 7-point Likert scale response format (0 = never, 6 = always), including 22 items that rate the three components of burnout: feelings of overwhelming emotional exhaustion (EE; sum of items 1, 2, 3, 6, 8, 14, 16, 20), depersonalization and detachment from the job (DP; sum of items 5, 10, 11, 15, 22), and lack of personal or professional accomplishment (PA; sum of items 4, 7, 9, 12, 17, 18, 19, 21) [25]. Burnout is indicated by high scores for EE and DP, and a low score for PA. The subjects were categorized on a severity scale according to the following cut-offs, previously used in an Italian survey on the COVID-19 pandemic on the general population and HCPs in Italy by Demartini et al. [26]:

- EE categorized into 0–18 (low), 19–26 (moderate), ≥27 (high);
- DP divided into 0–5 (low), 6–9 (moderate), ≥10 (high);
- PA classified into 0–33 (high), 34–39 (moderate), ≥ 40 (low) [26,27].

Fear of COVID-19 (FCV-19S) was used to evaluate an individual’s fear of COVID-19, using a seven-item scale [28] based on a five-item Likert-type scale (from 1 = strongly disagree to 5 = strongly agree). The total score ranges between 7 and 35, with a higher sum score indicating greater fear of COVID-19.

### 2.4. Statistical Analysis

Descriptive statistics (i.e., mean, standard deviation—SD, sample size) were performed to present the results for each measured variable. Statistical analyses included a paired or unpaired Student’s t-test (to determine if there is a *significant difference* between the means of two groups), χ^2^ test of independence (used to determine if there is an association between two or more categorical variables), and linear regression (to evaluate whether there is a relationship between the variables of interest). A comparison between more than two groups for total score and items was tested by using ANOVA (a factorial repeated measure of variance, within-subject variable: baseline vs. after training/between-subject factor and a Scheffe’s post hoc test: controls vs. experimental group). A significance level (alpha) of 0.05 was used. Statistical analysis was performed using Statview statistical software version 5.0.1 procedures (Abacus Concepts, Berkeley, CA, USA).

## 3. Results

### 3.1. Characteristics of the Participants

The distribution by sex, age and occupation of the subjects enrolled in the experimental group are shown in Table 1. The participants in the courses were mostly women, and their mean age was similar in the three groups. Specifically, the sample consisted of 27 nurses (26 women), 9 medical doctors (4 women), and other allied HCPs (11 women), who included 1 psychologist, 2 physiotherapists, 6 technicians, and 4 healthcare assistants all exposed to contact with patients. Work experience was 16.4 ± 7.7, 16.0 ± 7.7, and 17.1 ± 12.8 years (*p* = ns) in the three MBSR± groups, respectively.

Subjects belonging to the control groups displayed the following characteristics:

- control group I (CG I) = 15 subjects, mean age 42.1 ± 9.9, 12 women, 8 nurses, 5 physicians, 2 allied care professionals;
- control group II (CG II) = 2 subjects, mean age 42.2 ± 4.1, 1 woman, 1 nurse, 1 physician;
- control group III (CG III) = 11 subjects, mean age 39.2 ± 15.1, 10 women, 7 nurses, 2 physicians, 2 allied care professionals.

### 3.2. Psychological General Well-Being Index

#### 3.2.1. Baseline Characteristics and Differences between Groups at Baseline

Values of the six PGWBI domains and the total score at baseline in the three groups are reported in Table 2. The total scores in the three groups at baseline were 71.1 ± 14.6 (no distress), 60.3 ± 15.7 and 61.9 ± 14.9 (corresponding to a moderate distress), similar to those obtained in the control groups (59.5 ± 19.2 and 62.3 ± 12.9 in CG I and CG III, respectively).

No differences were found between baseline global score by gender, although there was a significant inverse correlation between baseline global score and aging (r = −0.3, *p* = 0.038).

#### 3.2.2. MBSR Effects on Psychological General Well-Being Index

Total scores significantly increased in Group I and III after MBSR training (77.2 ± 8.6 and 76.2 ± 11.6, respectively) (Table 2).

The number of participants exhibiting moderate or high levels of PGWBI, which significantly decreased after the MBSR course when compared to the baseline in Group III, are reported in Table 3.

**Figure 2:**
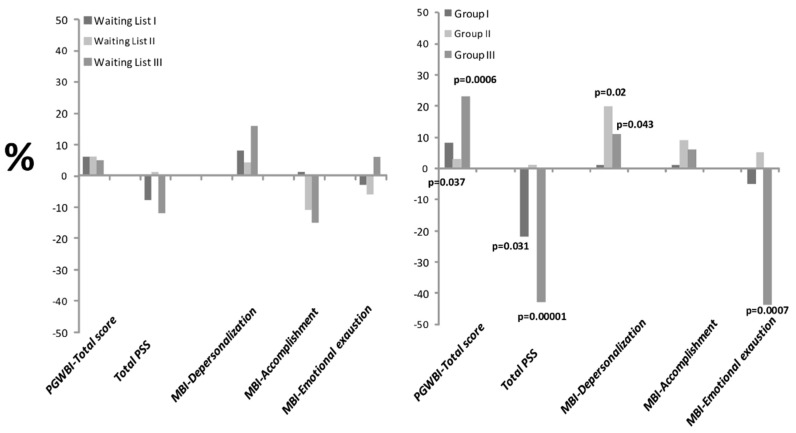
Percentage of change of PGWBI total score, total PSS, and the three MBI domains (depersonalization, accomplishment, and emotional exhaustion) between the two questionnaire administrations in the three WL and MSRB groups. Abbreviations: MBI: Maslach Burnout Inventory; PGWBI: Psychological General Well-Being Index; PSS: Perceived Stress Scale.

The values of the domains and total score did not significantly vary in the WL group after the MBSR course, as also shown by the percentage change before and after the MBSR training in Figure 2.

### 3.3. Perceived Stress Scale

#### 3.3.1. Baseline Characteristics and Differences between Groups at Baseline

The baseline total score did not differ by gender and age. Baseline total PSS scores, which did not vary among Groups I, II and III (corresponding to 18.0 ± 6.8, 20.9 ± 6.9 and 22.8 ± 4.6, respectively, *p* = ns), revealed moderate stress in HCPs (Table 2), with values comparable to those found in the control groups (23.5 ± 7.2 and 22.3 ± 5.9 in CGI and CG III, respectively). Table 3 also reports the number of participants showing moderate or high levels of PSS according to the total score categories.

#### 3.3.2. MBSR Effects on Perceived Stress Scale

After MBSR intervention, total PSS scores were characterized by significant decreased levels for Groups I and III (14.1 ± 5.6 and 13.3 ± 4.5 vs. baseline, respectively, reaching the lowest stress levels in Group III), but no change in Group II (20.3 ± 7.4 vs. baseline) (Table 2). The values of the total PSS did not significantly differ in the WL groups before and after the MBSR course, as shown by the percentage of change reported in Figure 2.

### 3.4. Maslach Burnout Inventory

#### 3.4.1. Baseline Characteristics and Differences between Groups at Baseline

No significant association was observed between aging and the three MBI domains at baseline. When considering gender differences, baseline DP resulted higher in men than in women (7.6 ± 6.1 vs. 3.9 ± 4.2, *p* = 0.04). Baseline EE did not change among Groups I, II and III (18.7 ± 7.9, 21.5 ± 7.8 and 16.4 ± 8.6, respectively, *p* = ns), indicating moderate levels of distress (Table 2), with values not significantly different to those observed in the control groups (21.8 ± 10.8 and 17.3 ± 9.3 in CG I and CG III, respectively).

The baseline DP was not different when evaluated between groups at baseline (4.9 ± 4.7, 3.2 ± 3.4 and 5.4 ± 5.8, respectively in Group I, II and III, *p* = ns), falling within the low category of distress, without displaying any significant difference to the values observed in CG I and CG II (3.9 ± 3.0 and 7.0 ± 5.3, respectively).

The baseline PA was similar in the three experimental groups (corresponding to 34.0 ± 6.4, 35.0 ± 7.8 and 34.1 ± 6.7, in Groups I, II and III, respectively, *p* = ns) and were not significantly different from CG I and CG II (37.1 ± 9.8 and 30.4 ± 5.9, respectively), revealing moderate stress (Table 2).

Table 3 shows the number of participants showing moderate or high levels according to the three principal MBI domain categories.

#### 3.4.2. MBSR Effects on Maslach Burnout Inventory

After the MBSR intervention, EE significantly decreased in Group III and DP significantly increased in Groups II and III, and CG III (Table 2 and Figure 2). The number of participants showing moderate or high levels according to the three principal MBI domain categories did not significantly vary after the MSRB course in any of the groups.

### 3.5. Fear of COVID-19 in Group III

#### 3.5.1. Baseline Characteristics and Differences between Groups at Baseline

Items 1 (I am most afraid of the coronavirus), 2 (It makes me uncomfortable to think about the coronavirus) and 5 (When watching news and stories about the coronavirus on social media, I become nervous or anxious) of the FCV-19S (belonging to the Emotional Fear Reactions) were high, with values more than 3 (Table 4). At baseline, the sample’s total FCV-19S mean score was 18.5 ± 4.3 (min–max 10–26). There was no significant difference between the baseline total scores for women (18.3 ± 4.4) and men (19.5 ± 5). Nonetheless, Item 1 was significantly higher in men; interestingly, despite the low number of subjects (n = 3), all men reported the maximum value (5 ± 0 vs. 3.6 ± 0.9 in women, *p* = 0.049). Aging was correlated with Item 1 (r = 0.8, *p* = 0.001), as well as with the total score (r = 0.6, *p* = 0.016) at baseline.

#### 3.5.2. MBSR Effects on “Fear of COVID-19”

After the MBSR intervention, values of Items 1 and 2 (emotional fear reactions), 6 (I cannot sleep because I’m worrying about getting the coronavirus), and 7 (My heart rates or palpitates when I think about getting the coronavirus), as symptomatic expressions of fear, as well as the total score, significantly decreased (Table 4).

## 4. Discussion

In the current study, the distress levels of HCPs before and after the pandemic were evaluated by PGWBI, PSS and MBI scoring. The effect of mindfulness training held before and in different periods during the pandemic on these parameters was also assessed. The “fear of COVID-19” questionnaire was also administered to HCPs enrolled after the spread of SARS-CoV-2 infection and participating in the last MBSR training course.

The main results observed are as follows:

1. In the baseline measures (T0), there was no statistical difference between the experimental and control groups, with all HCP groups exhibiting moderate stress;
2. Wellbeing (total PGWBI) decreased with aging. Moreover, FCV-19S positively correlated with age in HCPs;
3. MBSR was able to lower distress levels, except for increasing DP, while EE decreased in the MBSR groups trained after the pandemic onset (Group III);
4. HCPs had fear of COVID-19 (FCV-19S), but levels significantly decreased after MSRB training.

A previous assessment of distress in the Italian cardiology environmental workplace prior to the onset of COVID-19 showed work-related negative aspects, such as job strain, emotional fatigue and relational difficulties, which often create an unpleasant atmosphere and poor work performance [29]. The baseline level of distress found in our HCPs in the three groups falls within the moderate range, as previously reported in other Italian HCP cohorts [30]. While it is reasonable that the prolongation of the pandemic and the discomfort associated with the use of personal protective equipment, the difficulty of moving around and the limitation of essential physical needs may increase the distress, we did not observe any significant change in the baseline distress levels in the different analysis periods before and after the onset of the pandemic. Furthermore, the baseline moderate levels of distress observed in HCPs after the onset of the pandemic are also in agreement with other previously published data observed in Italian physicians during the COVID-19 period [24,31]. We did not notice any significant increase in baseline distress over time, which therefore appeared more affected by work-related distress than the pandemic in the study participants. Nonetheless, it will be important to monitor the individual’s long-term reaction to excessive and prolonged stress, which may lead to mental and physical exhaustion at a later phase of the pandemic in the single subject.

We observed that total PGWBI decreased and the level of COVID-19 fear increased with increasing age in HCPs. This is consistent with some previous findings, possibly because older HCPs were well aware that they were at increased risk of critical COVID-19 symptoms, due to the relationship between advanced age and mortality from COVID-19 complications [32,33]. A precedent study reported that younger HCPs were more fearful [34] but differences between enrolled subjects should be considered, as, unlike our study participants, very young workers with short or no previous work experience were included in this study, which may affect their ability to cope with emergencies.

Moreover, male HCPs showed significantly higher levels of DP than women, in accordance with previous observations showing that men tend to achieve higher DP scores compared to women, possibly due to their reduced willingness to express their emotions, and greater propensity to withdraw under stress [35].

It has been suggested that MBSR has potential to benefit clinicians in general by reducing distress and improving the well-being of HCPs [36,37,38]. Additionally, mindfulness practice has been associated with neurophysiological and neurobiological changes in key brain regions, linking this exercise to biological variations [39]. The number of intervention studies investigating the impact of mindfulness practices on individuals’ mental health in HCPs during the COVID-19 pandemic is limited. Nonetheless, MBSR interventions have been shown to reduce depression, stress, and anxiety symptoms during the COVID-19 pandemic [40,41,42]. PGWBI is a specific measure of psychological and emotional variables and covers both positive and negative dimensions. Except for vitality, all other PGWBI dimensions significantly improved at the end of the MBSR program in HCPs who took the course during the COVID-19 period (Group III), and total PSS also decreased. Thus, MBSR appears to be a practice that should be encouraged to reduce distress and fear, and improve wellbeing, especially in the context of a pandemic emergency.

EE greatly decreased after MBSR training in Group III. HCPs still seemed to be able to find some gratification from their work, which may be considered as a protective factor relevant to their own well-being, in emergency and stressful situations [43]. This finding is consistent with Karasek’s Demand–Control theory model, suggesting that HCPs with higher levels of work-related distress and heavier decision-making responsibilities were paradoxically deemed more powerful, more satisfied with their employment, and with lower disease levels [44]. Hence, HCPs who are directly and actively involved in work during the pandemic may enhance control of their activities, with greater chances of reaping rewards and coping with the stress related to the situation of uncertainty.

The increase in DP following MBSR may seem surprising. However, we should take into account that HCPs belonging to Groups II and III worked in stressful conditions during the pandemic. DP may therefore represent a possible maladaptive strategy to cope with stress. Interestingly, a recent systematic review evidenced that MBSR was less effective in reducing burnout (of which DP is a component), compared to the other aspects of psychological status that respond better to this training [45]. Indeed, other evidence suggests that burnout, although correlated with other components of distress (i.e., anxiety and depression), represents a definite and distinct construct [46]. Hence, burnout and/or its components may take a longer time to decrease than other psychological processes, or need the implementation of additional coping strategies to be reduced [47].

Interestingly, the baseline values of the FCS-19 items and total score in our HCPs were higher than those reported in the general Italian population [48]. On the other hand, the values of FCS-19 items and total score appeared lower than those found in the study of Soraci et al. [48] after MBSR training, suggesting the effectiveness of MBSR training to cope with the fear of COVID-19.

## 5. Strengths and Limitations

This study has some limitations. Firstly, data on daily meditation practice at home was collected on a self-reported adherence basis, which may not be accurate. Secondly, only a small sample of the control groups filled out the questionnaires at T1 (participation being voluntary for each phase of the study); thus, the WL groups included few participants, especially in CG II. However, we aimed to consider all participants in the control groups to have more subjects for the assessment of differences between groups at baseline. In addition, although WL groups included a very limited number of subjects, these small samples provide a comparison for the experimental groups to assess whether MBSR had an effect, and its impact.

In this study, MBSR training is able to improve well-being and reduce distress both in the pre- and post-COVID-19 period, with the exception of Group II. This group started the course in the pre-COVID period and completed the training after a few months (in the period following the first wave of the pandemic). Unfortunately, the participants were not re-administered a new baseline questionnaire; thus, it is unclear whether baseline distress was further increased over time during the first wave of the pandemic, so that MBSR training may have somehow reduced distress levels. However, the MBSR technique may not be very effective in reducing distress after the first traumatic wave of the pandemic or it may take a longer time to appreciate significant beneficial effects. Considering that the baseline values are always similar in all periods for all the control and experimental groups considered, we are inclined more toward the second scenario, probably resulting in an absence of differences following MBSR training after a very stressful and exhausting period characterized by the novelty of the emergency and a lack of organization, as with the first wave of COVID-19.

Finally, the study design would be stronger if it included a follow-up and if it involved a different comparison technique, rather than non-intervention. All these aspects may be considered to improve the design of future trials. Moreover, it would be interesting to match self-reported data obtained by questionnaires with measures of biological markers related to stress (i.e., salivary cortisol, oxidative stress/inflammatory biomarkers) to reinforce the psychological test with evidence of a biological response.

Nonetheless, the present results may be useful because there are few intervention studies available on how to effectively manage the psychological distress of HCPs, and to the best of our knowledge, none included groups that undergo MBSR training before and during the pandemic with HCPs and engaged in the same work environment. Thus, we hope our results may be of interest to anyone working in the healthcare system and especially professionals aiming to improve the well-being and mental status of HCPs.

The additional strengths of this study include a relatively large sample size divided into groups over time, the use of a battery of validated questionnaires, which made it possible to assess multicomponent entities of psycho-emotional status, and the application of a standardized MBSR intervention.

## 6. Conclusions

HCPs represent one of the groups most vulnerable to psychological distress. Moreover, SARS-CoV-2 has exposed HCPs to unprecedented levels of distress, as HCPs are (and feel) more at risk of being infected or unknowingly infecting others in their work, while the general population is protected from infection through quarantine and the use of preventive recommendations. Although these professionals represent a small percentage of the overall population, a significant percentage of COVID-19 cases have been encountered among HCPs [33]. For these reasons, monitoring the wellbeing of HCPs is urgent and can consequently better preserve the capacity of the healthcare system using tools that have proved effective, such as MBSR, especially in times of emergency such as the pandemic. The results reported in this study confirmed a significant positive impact of the intervention on certain psycho-emotional aspects, thus suggesting the MBSR technique as a potentially beneficial tool. In this context, subgroup analyses can highlight the importance of exploring the needs of potential participants, based on burden and responsibility, in order to select and implement the appropriate interventions.

## Figures and Tables

**Table 1:** Characteristics of MBSR course participants.

Variables	Group I	Group II	Group III	*p* Value
Number	17	18	15	
Women	15 (88)	15 (83)	12 (80)	ns
Age	43.4 (7.3)	44.8 (8.8)	45.2 (15.1)	ns
Occupation				
*Physicians*	2 (12)	4 (22)	3 (20)	ns
*Nurses*	9 (53)	9 (51)	9 (60)	
*Allied care professionals*	6 (35)	4 (22)	3 (20)	

Data are presented as mean (SD) or number (%). Abbreviations: MBSR: Mindfulness-Based Stress Reduction; ns: not significant.

**Table 2:** Values of psychometric domains and total scores in Groups I, II and III before and after the MBSR course.

Test	Group I Baseline	After MBSR	*p* Value	Group II Baseline	After MBSR	*p* Value	Group III Baseline	After MBSR	*p* Value
PGWBI *									
*Anxiety*	15.9 (4.8)	17.6 (2.6)	ns	13.4 (4.4)	14.5 (4.9)	ns	13.7 (4.1)	18.0 (3.4)	0.0005
*Depressed mood*	12.5 (2.9)	12.9 (1.2)	ns	10.8 (3.4)	10.7 (3.6)	ns	10.5 (2.0)	12.4 (1.7)	0.001
*Positive wellbeing*	11.2 (4.1)	12.4 (2.6)	ns	9.2 (3.6)	9.5 (4.5)	ns	9.5 (3.2)	11.9 (3.1)	0.005
*Self-control*	11.3 (2.4)	11.4 (2.1)	ns	8.9 (3.3)	8.7 (3.6)	ns	8.7 (3.1)	11.7 (2.1)	0.002
*General health*	10.1 (1.5)	12.1 (1.7)	0.0004	9.2 (3.2)	9.2 (3.7)	ns	9.5 (3.0)	11.4 (2.1)	0.008
*Vitality*	10.0 (2.2)	10.8 (1.9)	ns	8.9 (1.7)	9.3 (2.1)	ns	10.0 (2.1)	10.9 (1.4)	ns
*Total score*	71.1 (14.6)	77.2 (8.6)	0.04	60.3 (15.7)	61.8 (18.2)	ns	61.9 (14.9)	76.2 (11.6)	0.0006
PSS									
*Total PSS*	18.0 (6.8)	14.1 (5.6)	0.03	20.9 (6.9)	20.3 (7.4)	ns	22.8 (4.6)	13.3 (4.5)	0.0001
MBI									
*Depersonalization*	4.9 (4.7)	5.5 (5.5)	ns	3.2 (3.4)	4.0 (4.3)	0.02	5.4 (5.8)	6.1 (6.9)	0.04
*Accomplishment*	34.0 (6.4)	34.1 (7.6)	ns	35.0 (7.8)	37.9 (4.5)	ns	34.1 (6.7)	35.6 (6.5)	ns
*Emotional exhaustion*	18.7 (7.9)	18.3 (7.2)	ns	21.5 (7.8)	21.6 (10.2)	ns	16.4 (8.6)	8.6 (7.2)	0.0007

Data are presented as mean (SD). Abbreviations: MBI: Maslach Burnout Inventory; MBSR: Mindfulness-Based Stress Reduction; ns: not significant; PGWBI: Psychological General Well-Being Index; PSS: Perceived Stress Scale. * Higher scores indicate a better PGWBI.

**Table 3:** Number of subjects with moderate or high levels of the distress domains in Groups I, II and III before and after the MBSR course.

Test		Group I Baseline	After MBSR	*p* Value	Group II Baseline	After MBSR	*p* Value	Group III Baseline	After MBSR	*p* Value
PGWBI										
*Total score*	Moderate	1	2	ns	6	4	ns	3	2	0.03
	High	3	0		6	3		5	0	
PSS										
*Total PSS*	Moderate	11	9	0.049	13	10	ns	12	8	0.026
	High	3	0		3	2		2	0	
MBI										
*Depersonalization*	Moderate	6	6	ns	1	3	ns	3	3	ns
	High	2	2		2	2		3	3	
*Accomplishment*	Moderate	3	3	ns	4	4	ns	4	5	ns
	High	3	5		4	4		4	4	
*Emotional exhaustion*	Moderate	8	8	ns	7	5	ns	3	0	ns
	High	1	2		5	2		3	1	

Data are presented as numbers (SD). Abbreviations: MBI: Maslach Burnout Inventory; MBSR: Mindfulness-Based Stress Reduction; ns: not significant; PGWBI: Psychological General Well-Being Index; PSS: Perceived Stress Scale.

**Table 4:** Items and total FCS-19 in Group III before and after the MBSR course.

	Baseline		Post MBSR Course		
	Mean	SD	Mean	SD	*p* Value
1. I am most afraid of the coronavirus	3.8	1.0	3.0	1.3	0.015
2. It makes me uncomfortable to think about the coronavirus	3.7	0.6	2.9	1.3	0.017
3. My hands become clammy when I think about the coronavirus	1.6	0.9	1.2	0.5	ns
4. I am afraid of losing my life because of the coronavirus	2.4	0.8	2.0	1.0	ns
5. When watching news and stories about the coronavirus on social media, I become nervous or anxious	3.2	0.9	2.6	1.2	ns
6. I cannot sleep because I’m worrying about getting the coronavirus	1.8	0.9	1.2	0.5	0.04
7. My heart rates or palpitates when I think about getting the coronavirus	2.0	1.0	1.5	0.8	0.049
Total score	18.5	4.3	14.4	5.1	0.009

Abbreviations: MBSR: Mindfulness-Based Stress Reduction; ns: not significant.

## Data Availability

The data that support the findings of this study are available upon request from the authors for research purposes.

## Abbreviations

DP	Depersonalization
EE	Emotional exhaustion
FCV-19S	Fear of COVID-19 Scale
HCP	Healthcare professional
MBI	Maslach Burnout Inventory
MBSR	Mindfulness-Based Stress Reduction
PA	Personal or professional accomplishment
PGWBI	Psychological General Well-Being Index
PS	Perceived Stress
PSS	Perceived Stress Scale
WL	Waiting list

## References

[1] Pappa S., Ntella V., Giannakas T., Giannakoulis V.G., Papoutsi E., Katsaounou P. (2020). Prevalence of depression, anxiety, and insomnia among healthcare workers during the COVID-19 pandemic: A systematic review and meta-analysis. Brain Behav. Immun..

[2] Vindegaard N., Benros M.E. (2020). COVID-19 pandemic and mental health consequences: Systematic review of the current evidence. Brain Behav. Immun..

[3] Luo M., Guo L., Yu M., Jiang W., Wang H. (2020). The psychological and mental impact of coronavirus disease 2019 (COVID-19) on medical staff and general public—A systematic review and meta-analysis. Psychiatry Res..

[4] Di Tella M., Romeo A., Benfante A., Castelli L. (2020). Mental health of healthcare workers during the COVID-19 pandemic in Italy. J. Eval. Clin. Pract..

[5] Lasalvia A., Bonetto C., Porru S., Carta A., Tardivo S., Bovo C., Ruggeri M., Amaddeo F. (2020). Psychological impact of COVID-19 pandemic on healthcare workers in a highly burdened area of north-east Italy. Epidemiol Psychiatr. Sci..

[6] Fournier A., Laurent A., Lheureux F., Ribeiro-Marthoud M.A., Ecarnot F., Binquet C., Quenot J.P. (2022). Impact of the COVID-19 pandemic on the mental health of professionals in 77 hospitals in France. PLoS ONE.

[7] Hummel S., Oetjen N., Du J., Posenato E., Resende de Almeida R.M., Losada R., Ribeiro O., Frisardi V., Hopper L., Rashid A. (2021). Mental Health Among Medical Professionals During the COVID-19 Pandemic in Eight European Countries: Cross-sectional Survey Study. J. Med. Internet Res..

[8] Botha E., Gwin T., Purpora C. (2015). The effectiveness of mindfulness based programs in reducing stress experienced by nurses in adult hospital settings: A systematic review of quantitative evidence protocol. JBI Database Syst. Rev. Implement Rep..

[9] Dharmawardene M., Givens J., Wachholtz A., Makowski S., Tjia J. (2016). A systematic review and meta-analysis of meditative interventions for informal caregivers and health professionals. BMJ Support Palliat. Care..

[10] McConville J., McAleer R., Hahne A. (2017). Mindfulness Training for Health Profession Students-The Effect of Mindfulness Training on Psychological Well-Being, Learning and Clinical Performance of Health Professional Students: A Systematic Review of Randomized and Non-randomized Controlled Trials. Explore.

[11] Lomas T., Medina J.C., Ivtzan I., Rupprecht S., Eiroa-Orosa F.J. (2018). A systematic review of the impact of mindfulness on the well-being of healthcare professionals. J. Clin. Psychol..

[12] Spinelli C., Wisener M., Khoury B. (2019). Mindfulness training for healthcare professionals and trainees: A meta-analysis of randomized controlled trials. J. Psychosom. Res..

[13] Rodriguez-Vega B., Palao Á., Muñoz-Sanjose A., Torrijos M., Aguirre P., Fernández A., Amador B., Rocamora C., Blanco L., Marti-Esquitino J. (2020). Implementation of a Mindfulness-Based Crisis Intervention for Frontline Healthcare Workers During the COVID-19 Outbreak in a Public General Hospital in Madrid, Spain. Front. Psychiatry.

[14] Upadhyay P., Narayanan S., Khera T., Kelly L., Mathur P.A., Shanker A., Novack L., Sadhasivam S., Hoffman K.A., Pérez-Robles R. (2021). Perceived stress, resilience, well-being, and COVID 19 response in Isha yoga practitioners compared to matched controls: A research protocol. Contemp. Clin. Trials Commun..

[15] Faragher E.B., Cass M., Cooper C.L. (2005). The relationship between job satisfaction and health: A meta-analysis. Occup. Env. Med..

[16] Klockner K., Crawford C., Craigie M., Tsai L., Hegney D. (2021). A qualitative exploration of a mindful resiliency program for community healthcare providers. Nurs. Health Sci..

[17] Kabat-Zinn J. (2003). Mindfulness-based interventions in context: Past, present, and future. Clin. Psychol. Sci. Pract..

[18] Islam M., George P., Sankaran S., Su Hui J.L., Kit T. (2021). Impact of COVID-19 on the mental health of healthcare workers in different regions of the world. BJPsych Open..

[19] Zhang Q., Dong G., Meng W., Chen Z., Cao Y., Zhang M. (2022). Perceived Stress and Psychological Impact Among Healthcare Workers at a Tertiaty Hospital in China During the COVID-19 Outbreak: The Moderating Role of Resilience and Social Support. Front. Psychiatry.

[20] Shbeer A., Ageel M. (2022). Assessment of Occupational Burnout among Intensive Care Unit Staff in Jazan, Saudi Arabia, Using the Maslach Burnout Inventory. Crit. Care Res. Pract..

[21] Alyami H., Krägeloh C.U., Medvedev O.N., Alghamdi S., Alyami M., Althagafi J., Lyndon M., Hill A.G. (2022). Investigating Predictors of Psychological Distress for Healthcare Workers in a Major Saudi COVID-19 Center. Int. J. Environ. Res. Public Health.

[22] Dupuy H.J., Wenger N.K., Mattson M.E., Furberg C.D., Elinson J. (1984). Psychological General Well-Being Index (PGWB). Assessment of Quality of Life in Clinical Trials of Cardiovascular Therapies.

[23] Cohen S., Kamarck T., Mermelstein R. (1983). A global measure of perceived stress. J. Health Soc. Behav..

[24] Ozen G., Zanfardino A., Ozen G., Acan B., Piscopo A., Casaburo F., Gicchino F., Confetto S., Troncone A., Iafusco D. (2021). Comparison of emotional approaches of medical doctors against COVID-19 pandemic: Eastern and Western Mediterranean countries. Int. J. Clin. Pract..

[25] Maslach‚ C., Jackson‚ S. (1981). MBI: Maslach Burnout Inventory Manual.

[26] Demartini B., Nisticò V., D’Agostino A., Priori A., Gambini O. (2020). Early Psychiatric Impact of COVID-19 Pandemic on the General Population and Healthcare Workers in Italy: A Preliminary Study. Front. Psychiatr..

[27] Maslach C., Jackson S., Leiter M. (1997). The Maslach Burnout Inventory Manual.

[28] Ahorsu D.K., Lin C.Y., Imani V., Saffari M., Griffiths M.D., Pakpour A.H. (2020). The Fear of COVID-19 Scale: Development and Initial Validation. Int. J. Ment. Health Addict..

[29] Majani G., Di Tano G., Giardini A., De Maria R., Russo G., Maestri R., Marini M., Milli M., Aspromonte N. (2016). Prevalence of job-related distress and satisfaction in a nationwide cardiology setting: The IANUS—Italian cardiologists’ Undetected distress Study. J. Cardiovasc. Med. (Hagerstown).

[30] Mattei A., Fiasca F., Mazzei M., Necozione S., Bianchini V. (2017). Stress and Burnout in Health-Care Workers after the 2009 L’Aquila Earthquake: A Cross-Sectional Observational Study. Front. Psychiatr..

[31] Rossi R., Socci V., Pacitti F., Di Lorenzo G., Di Marco A., Siracusano A., Rossi A. (2020). Mental Health Outcomes Among Frontline and Second-Line Health Care Workers During the Coronavirus Disease 2019 (COVID-19) Pandemic in Italy. JAMA Netw. Open..

[32] Kabasakal E., Özpulat F., Akca A., Özcebe L.H. (2021). COVID-19 fear and compliance in preventive measures precautions in workers during the COVID-19 pandemic. Int. Arch. Occup. Environ. Health.

[33] Troisi A., Nanni R.C., Riconi A., Carola V., Di Cave D. (2021). Fear of COVID-19 among Healthcare Workers: The Role of Neuroticism and Fearful Attachment. J. Clin. Med..

[34] Rossi R., Socci V., Pacitti F., Mensi S., Di Marco A., Siracusano A., Di Lorenzo G. (2020). Mental Health Outcomes Among Healthcare Workers and the General Population During the COVID-19 in Italy. Front. Psychol..

[35] Maslach C., Schaufeli W.B., Leiter M.P. (2001). Job burnout. Annu. Rev. Psychol..

[36] Watson T., Walker O., Cann R., Varghese A.K. (2021). The benefits of mindfulness in mental healthcare professionals. F1000Research.

[37] Fortney L., Luchterhand C., Zakletskaia L., Zgierska A., Rakel D. (2013). Abbreviated mindfulness intervention for job satisfaction, quality of life, and compassion in primary care clinicians: A pilot study. Ann. Fam. Med..

[38] Montero-Marin J., Tops M., Manzanera R., Piva Demarzo M.M., Álvarez de Mon M., García-Campayo J. (2015). Mindfulness, Resilience, and Burnout Subtypes in Primary Care Physicians: The Possible Mediating Role of Positive and Negative Affect. Front. Psychol..

[39] Treadway M.T., Lazar S.W., Didonna F. (2009). The neurobiology of mindfulness. Clinical Handbook of Mindfulness.

[40] Weis R., Ray S.D., Cohen T.A. (2020). Mindfulness as a way to cope with COVID-19-related stress and anxiety. Couns. Psychother. Res..

[41] Matiz A., Fabbro F., Paschetto A., Cantone D., Paolone A.R., Crescentini C. (2020). Positive Impact of Mindfulness Meditation on Mental Health of Female Teachers during the COVID-19 Outbreak in Italy. Int. J. Environ. Res. Public Health.

[42] Zhang H., Zhang A., Liu C., Xiao J., Wang K. (2021). A Brief Online Mindfulness-Based Group Intervention for Psychological Distress Among Chinese Residents During COVID-19: A Pilot Randomized Controlled Trial. Mindfulness.

[43] Bonetti L., Tolotti A., Valcarenghi D., Pedrazzani C., Barello S., Ghizzardi G., Graffigna G., Sari D., Bianchi M. (2019). Burnout Precursors in Oncology Nurses: A Preliminary Cross-Sectional Study with a Systemic Organizational Analysis. Sustainability.

[44] Theorell T., Karasek R.A. (1996). Current issues relating to psychosocial job strain and cardiovascular disease research. J. Occup. Health Psychol..

[45] Kriakous S.A., Elliott K.A., Lamers C., Owen R. (2021). The Effectiveness of Mindfulness-Based Stress Reduction on the Psychological Functioning of Healthcare Professionals: A Systematic Review. Mindfulness.

[46] Awa W.L., Plaumann M., Walter U. (2010). Burnout prevention: A review of intervention programs. Patient Educ. Couns..

[47] Crowder R., Sears A. (2017). Building resilience in social workers: An exploratory study on the impacts of a mindful-ness-based intervention. Aus. Soc. Work.

[48] Soraci P., Ferrari A., Abbiati F.A., Del Fante E., De Pace R., Urso A., Griffiths M.D. (2020). Validation and Psychometric Evaluation of the Italian Version of the Fear of COVID-19 Scale. Int. J. Ment. Health Addict..

